# Conducting a Virtual Clinical Trial in HER2-Negative Breast Cancer Using a Quantitative Systems Pharmacology Model With an Epigenetic Modulator and Immune Checkpoint Inhibitors

**DOI:** 10.3389/fbioe.2020.00141

**Published:** 2020-02-25

**Authors:** Hanwen Wang, Richard J. Sové, Mohammad Jafarnejad, Sondra Rahmeh, Elizabeth M. Jaffee, Vered Stearns, Evanthia T. Roussos Torres, Roisin M. Connolly, Aleksander S. Popel

**Affiliations:** ^1^Department of Biomedical Engineering, Johns Hopkins University School of Medicine, Baltimore, MD, United States; ^2^Department of Oncology, The Sidney Kimmel Cancer Center, Johns Hopkins University School of Medicine, Baltimore, MD, United States; ^3^Viragh Center for Pancreatic Clinical Research and Care, Bloomberg Kimmel Institute for Immunotherapy, Johns Hopkins University School of Medicine, Baltimore, MD, United States

**Keywords:** immuno-oncology, immune checkpoint inhibitor, computational model, systems biology, epigenetic modulator, quantitative systems pharmacology, virtual clinical trial

## Abstract

The survival rate of patients with breast cancer has been improved by immune checkpoint blockade therapies, and the efficacy of their combinations with epigenetic modulators has shown promising results in preclinical studies. In this prospective study, we propose an ordinary differential equation (ODE)-based quantitative systems pharmacology (QSP) model to conduct an *in silico* virtual clinical trial and analyze potential predictive biomarkers to improve the anti-tumor response in HER2-negative breast cancer. The model is comprised of four compartments: central, peripheral, tumor, and tumor-draining lymph node, and describes immune activation, suppression, T cell trafficking, and pharmacokinetics and pharmacodynamics (PK/PD) of the therapeutic agents. We implement theoretical mechanisms of action for checkpoint inhibitors and the epigenetic modulator based on preclinical studies to investigate their effects on anti-tumor response. According to model-based simulations, we confirm the synergistic effect of the epigenetic modulator and that pre-treatment tumor mutational burden, tumor-infiltrating effector T cell (Teff) density, and Teff to regulatory T cell (Treg) ratio are significantly higher in responders, which can be potential biomarkers to be considered in clinical trials. Overall, we present a readily reproducible modular model to conduct *in silico* virtual clinical trials on patient cohorts of interest, which is a step toward personalized medicine in cancer immunotherapy.

## Introduction

Although in clinical trials immunotherapies using anti-PD-1 and anti-PD-L1 antibodies and their combinations with other types of therapies have improved the overall response rate and progression-free survival rate in patients with breast cancer, more than half of patients developed progressive disease (Emens, 2018). To improve the efficacy of checkpoint inhibitors, multiple strategies are being developed to facilitate antigen release, T cell activation and homing, and improve tumor microenvironment, such as cancer vaccines and anti-OX40 antibody therapy (Hu-Lieskovan and Ribas, 2017). In March 2019, the Food and Drug Administration (FDA) granted an accelerated approval for the immunotherapy anti-PD-L1 drug, atezolizumab, in combination with chemotherapy drug, nanoparticle albumin–bound paclitaxel (nab-paclitaxel), for the initial treatment of some women with advanced triple-negative breast cancer (TNBC). Among the ongoing clinical trials in breast cancer, a phase I trial using a triple combination of anti-CTLA-4 and anti-PD-1 antibodies, and a small-molecule epigenetic modulator, entinostat, tests safety, efficacy and impact on the ratio of tumor-specific effector T cell (Teff) to regulatory T cell (Treg) (NCT02453620).

Entinostat, also called MS-275, was originally developed as an antitumor agent, which inhibits histone deacetylases (HDAC) and induces a shift of cell cycle from S phase to G_1_ phase (Saito et al., 1999). There is emerging evidence that it can alter the immune-suppressive microenvironment in the tumor (Connolly et al., 2017; Christmas et al., 2018). Preclinical studies also suggest that the alteration of the tumor microenvironment can improve the efficacy of checkpoint blockade therapy (Kato et al., 2014; Pili et al., 2017). In an *in vivo* experiment by Kim et al., the addition of entinostat significantly reduced tumor volume in 4T1 and CT26 mouse models under anti-PD-1 and anti-CTLA-4 antibody treatment (Kim et al., 2014). In a recent study, combining entinostat with anti-PD-1, anti-CTLA-4, or both significantly improved tumor-free survival in the HER-2/neu transgenic breast cancer mouse model (Christmas et al., 2018).

The success of entinostat treatment in preclinical studies has also drawn the attention to myeloid-derived suppressor cells (MDSCs) in the breast tumor microenvironment. In breast cancer patients, MDSC level is correlated to cancer stages and metastasis (Gonda et al., 2017). As a major contributor of the immune suppression in peripheral lymphoid tissues, the inhibitory effect of MDSCs is also found to be augmented in the tumor microenvironment, such as Treg expansion and inhibition of Teff functions (Kumar et al., 2016). Although a number of mechanisms are considered to be the potential causes of their inhibitory effects, recent studies suggest that Arginase I (Arg-I) and nitric oxide (NO) are the major immune-suppressive molecules secreted by MDSCs (Alotaibi et al., 2018; Park et al., 2018; Sheikhpour et al., 2018). Due to their significant inhibition of adaptive immune response in the tumor microenvironment, MDSCs have been suggested as a target for breast cancer treatment (Markowitz et al., 2013).

Besides the significant reduction of tumor volume, entinostat is also suggested to alter MDSC levels both in blood and in the tumor microenvironment; to change the proportions of T cell subsets; and to increase tumor sensitivity to CTL-mediated lysis (Kim et al., 2014; Gameiro et al., 2016; Orillion et al., 2017; Christmas et al., 2018). Experiments detected a significant reduction of tumor-infiltrating FoxP3^+^ Treg and granulocytic MDSC (G-MDSCs) (vs. monocytic MDSC, M-MDSC) in mice receiving entinostat treatment (Kim et al., 2014; Christmas et al., 2018). A separate preclinical study also observed the enhanced antitumor immune response with significantly decreased FoxP3^+^ expression in circulating Tregs and increased tumor-infiltrating G-MDSCs in syngeneic mouse cancer models under entinostat and anti-PD-1 antibody treatment (Orillion et al., 2017). Although preclinical studies have provided somewhat controversial conclusions on how entinostat alters the composition of T cell subsets and MDSCs in the tumor microenvironment, they all suggest that entinostat reverses the inhibitory effects of MDSCs (Kim et al., 2014; Orillion et al., 2017; Christmas et al., 2018).

Due to the promising efficacy of entinostat treatment in preclinical studies, the effects of entinostat were investigated with exemestane/placebo in locally advanced or metastatic hormone receptor-positive breast cancer (Yardley et al., 2013; Tomita et al., 2016; Yeruva et al., 2018). In a phase II trial, both progression-free survival and overall survival rates were significantly higher in the entinostat-treated cohort. These results have led to a phase III trial (E2112, NCT02115282) that aims to validate the preclinical and clinical evidence supporting the role of HDAC inhibitors in improving outcomes for patients with advanced breast cancer (Yeruva et al., 2018). In addition, the synergistic effect of entinostat in combination therapy with anti-PD-1 antibody, nivolumab, has been reported in melanoma patients. Patients who had stable or progressive disease in previous checkpoint blockade therapy were converted to responders with entinostat treatment (Agarwala et al., 2018).

Since the characteristics of patients who are likely to benefit from epigenetic modulation are still unknown, we propose an expanded quantitative systems pharmacology (QSP) model based on our previous steps (Jafarnejad et al., 2019; Milberg et al., 2019; Wang et al., 2019). It is built with a detailed MDSC module and pharmacokinetics and pharmacodynamics of entinostat, to investigate the effect of entinostat and its combination with nivolumab and ipilimumab by conducting an *in silico* virtual clinical trial. Virtual clinical trials aim to generate virtual patient cohorts with physiologically plausible parameters and predict efficacies of treatments of interest using *in silico* simulations with a QSP model (Allen et al., 2016; Cheng et al., 2017; Rieger et al., 2018). Due to the heterogeneity of patient cohorts enrolled in clinical trials and wide range of treatment strategies, *in silico* simulations using a virtual patient cohort that resembles the desired clinical population can provide insights into the potential therapeutic outcome even before the therapy begins. In this study, we will conduct an *in silico* virtual clinical trial to explore the effects of different factors and patients’ characteristics prospectively, ahead of the results of the ongoing clinical trial (NCT02453620).

## Materials and Methods

### Model Overview

The proposed QSP model has a general structure similar to the model introduced in our previous studies (Jafarnejad et al., 2019; Wang et al., 2019). It comprises four compartments: central, peripheral, tumor, and tumor-draining lymph node (TDLN). The central and the peripheral compartments represent the total volume of blood and peripheral tissues, respectively. The TDLN compartment represents a lumped lymph node assuming that the antibody and T cell activation is evenly distributed among a number of TDLNs. The tumor compartment represents the total tumor volume, which is calculated at each time step as the addition of the total volume of proliferating and dead cancer cells, T cells, and other cells and tumor interstitium. Tumor diameter is calculated using total tumor volume assuming a spherical tumor, which is an estimate of mean lesion size for each virtual patient.

**FIGURE 1:**
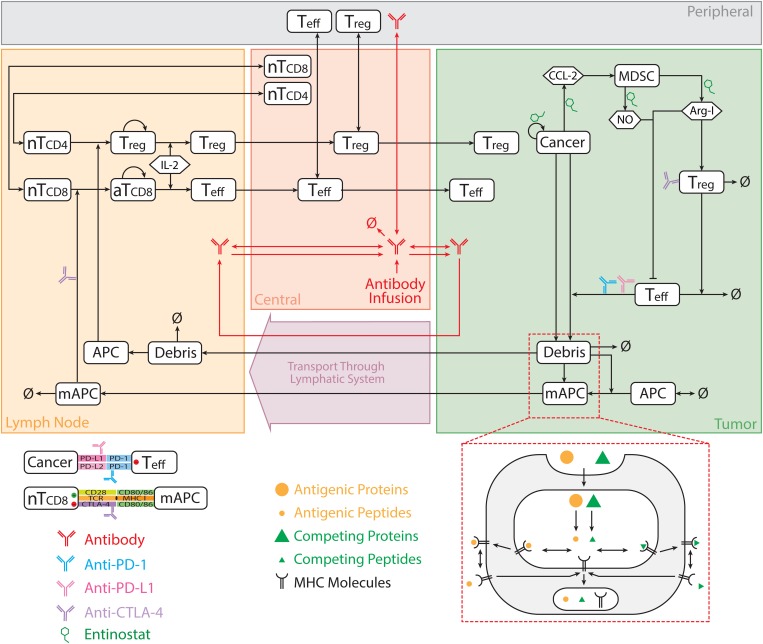
Model diagram. The model is comprised of four compartments: central, peripheral, tumor, and tumor-draining lymph node, which describe cycles of immune activation in lymph nodes, T cell trafficking to the tumor, killing of cancer cells, immune evasion, and antigen release and lymphatic transport. Anti-CTLA-4 antibody blocks interaction between CD80/86 and CTLA-4 on mAPC and naïve T cell, respectively, in lymph nodes, and induces ADCC-mediated Treg depletion in the tumor. Anti-PD-1 antibody blocks interaction between PD-1 and PD-L1/2 on Teff and cancer cell, respectively, in the tumor. nT, naïve T cell; aT, activated T cell; NO, nitric oxide; Arg-I, arginase I; Treg, regulatory T cell; Teff, effector T cell; mAPC, mature antigen presenting cell. The figure is adapted from Jafarnejad et al. (2019).

The model comprises multiple modules, each of which describes the dynamics of one of the major species (i.e., effector T cells, regulatory T cells, MDSCs, cancer cells, antigen-presenting cells (APCs), antigens, checkpoint ligands and receptors, and therapeutic agents); each module is built separately using MATLAB (MathWorks, Natick, MA, United States) scripts. The modular structure of this model greatly facilitates modifications and expansions for future applications. The model used in this study comprises eight modules, 210 parameters, 120 ordinary differential equations (ODEs) and 39 algebraic equations, which are implemented using the SimBiology toolbox in MATLAB. The dynamics of the major species in the model are illustrated in Figure 1. Full lists of model parameters, reactions, algebraic equations, and cellular and molecular species are included in the Supplementary Tables S1–S6.

### Pharmacokinetics and Pharmacodynamics (PK/PD) of Entinostat

**FIGURE 2:**
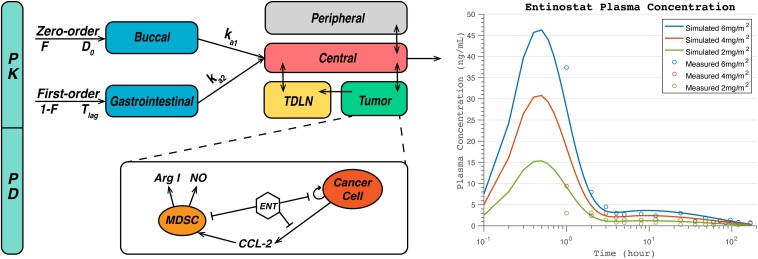
Diagram of pharmacokinetic/pharmacodynamic (PK/PD) module for entinostat **(A)** and simulated and measured plasma concentration at doses of 2, 4, 6 mg/m^2^ **(B)**. A fraction of dose, F, is immediately absorbed by the patients via zero-order buccal absorption over a time duration D_0_ into the buccal compartment, and the rest of the dose, 1-F, is absorbed after a time period T_lag_ via first-order gastrointestinal (GI) absorption into the gastrointestinal compartment. The drug in buccal and gastrointestinal compartments are then absorbed into the central compartment via first-order absorption, and transported into the peripheral and the tumor compartments via passive diffusion. For pharmacodynamics of entinostat, it inhibits nitric oxide (NO) and arginase I (Arg-I) production by myeloid-derived suppressor cells (MDSCs), CCL2 production by cancer cells, Arg-I activity, and cancer cell proliferation.

Since there is no published population-based pharmacokinetic model for entinostat, we propose a model structure for this oral-administered drug based on four published clinical PK studies of entinostat, and the PK parameters are optimized using data reported in these studies (Ryan et al., 2005; Gojo et al., 2007; Kummar et al., 2007; Gore et al., 2008). Parameter optimization is performed using pattern search in the MATLAB Global Optimization Toolbox. Multi-compartment PK model structures are tested using similar methods from Gasthuys et al. (2018) and the final diagram of our proposed PK model structure is demonstrated in Figure 2A. As shown in the figure, a portion of the dose is immediately absorbed by the patients via zero-order buccal absorption over a time duration D_0_ into the buccal compartment, and the rest of the dose is absorbed after a time period T_lag_ via first-order gastrointestinal absorption into the gastrointestinal compartment. The drug in buccal and gastrointestinal compartments are then absorbed into the central compartment via first-order absorption and diffuse into the peripheral and the tumor compartments. For pharmacodynamics of entinostat, it is known to induce cell cycle arrest in cancer cells, reduce their viability, and significantly reduce the level of immune-suppressive cytokines in the tumor (Lee et al., 2001; Bouchain et al., 2003; Choo et al., 2010, 2013; Ryu et al., 2019). In the current module, we assume that the major mechanisms of action for entinostat are inhibitions of cancer cell proliferation and production of monocyte chemoattractant protein-1 (MCP-1/CCL2) and nitric oxide, by which entinostat has shown to reverse the immune-suppressive effects of MDSCs (Kim et al., 2014; Orillion et al., 2017; Christmas et al., 2018). The PD parameters of entinostat are listed in Supplementary Table S7, and its anti-proliferative effect on breast cancer cells is shown in Supplementary Figure S1. Since the effect of entinostat on MDSC level and T cell subsets are still under investigation, it is assumed not to have direct impact on any species other than cancer cells in the model (Kim et al., 2014; Orillion et al., 2017; Christmas et al., 2018). In addition, the PK/PD of checkpoint blockade antibodies are implemented using the same equations as in our previous model based on published clinical data (Feng et al., 2014; Bajaj et al., 2017; Wang et al., 2019).

### MDSC Module

In addition to the mechanisms from our previous model (Jafarnejad et al., 2019), a new MDSC module is implemented to describe the immune-suppressive mechanisms of MDSC including Treg expansion and inhibition of effector T cell function. MDSCs are recruited into the tumor compartment by CCL2 secreted by cancer cells in addition to a baseline recruitment, and the predicted CCL2 expression and migration indices are fitted to TNBC data (Supplementary Figure S3; Huang et al., 2007; Dutta et al., 2018). The factors secreted by MDSCs as the major contributors of their inhibitory effects are assumed to be Arg-I and NO, whose expression rates are estimated based on *in vitro* experiments on breast cancer cells (Supplementary Figure S2; Serafini et al., 2008). Since only the enzymatic activity of Arg-I is measured in enzyme unit, mU, we use mU as a placeholder of Arg-I concentration in the model, assuming that the protein concentration is proportional to the enzymatic activity. The unit of its production rate is then set to be mU^∗^(microliter)/cell/day to estimate the amount of Arg-I produced by MDSCs per day. The unit of production rates of NO and CCL2 is set to be nanomole/cell/day. While both Arg-I and NO inhibit cytotoxic killing of cancer cells by effector T cells, only Arg-I facilitates Treg expansion in the tumor (Serafini et al., 2008). The effective concentrations are estimated based on *in vitro* experiments and listed in Supplementary Table S7 with references.

### Mechanisms of Anti-CTLA-4 Activity

#### Checkpoint Activity of CTLA-4

Dynamics of CTLA-4 related checkpoint molecules are modeled based on the previously published model by Jansson et al. (2005), and cross-arm binding of monoclonal anti-CTLA-4 antibody to CTLA-4 is incorporated similar to that of anti-PD-1 antibody to PD-1 in our previous model (Harms et al., 2014; Jafarnejad et al., 2019). Briefly, CD28 and CTLA-4 on naïve T cells are assumed to bind CD80 and CD86 on APCs. CD28 is a co-stimulatory signal that enhances the activation of naïve T cells resulting in higher levels of proliferating T cells. Higher affinity of CTLA-4 for CD80/CD86 results in the depletion of CD28 ligands for T cell activation, and blockade of CTLA-4 restores ligand availability for CD28 that leads to enhanced T cell activation and proliferation. Furthermore, the binding of CD80 on APC to PD-L1 on T cells was included to compete with the interactions of PD-1 and CTLA-4 related axes (Sugiura et al., 2019). All the reactions are assumed to happen in the two-dimensional synapse compartment between the respective cells and the details of the reactions are included in the Supplementary Material. The biochemical parameters of this module are mostly measured experimentally and reported in the literature (Jansson et al., 2005).

#### Anti-CTLA4-Mediated Antibody-Dependent Cellular Cytotoxicity (ADCC)

In addition to the checkpoint activity of CTLA-4, ADCC is shown to be a potential mechanism of action for antibodies targeting CTLA-4 (Arce Vargas et al., 2018). Regulatory T cells express higher levels of CTLA-4 compared to effector T cells, and anti-CTLA-4 antibodies enhance Treg depletion through ADCC (Arce Vargas et al., 2018). It should be noted that the importance of this mechanism in human has been questioned by clinical observations (Sharma et al., 2019). In this model, anti-CTLA4-mediated ADCC is incorporated as Treg depletion through binding between the anti-CTLA-4 antibody and CTLA-4 on Treg in the tumor. The maximal Treg depletion rate is estimated based on *in vitro* experiments (Richards et al., 2008).

### Simulation Settings

Although the model is used to simulate PK/PD for all the immune checkpoint antibodies (i.e., anti-CTLA-4, anti-PD-1, and anti-PD-L1) and entinostat in monotherapy and combination therapies, we focus on the particular clinical trial (NCT02453620), in which nivolumab and entinostat are administered to 26 HER2-negative breast cancer patients with or without ipilimumab. The breast cancer-specific parameters, including cancer cell diameter, the number of tumor-draining lymph nodes, tumor growth rate, volume fractions, and steady-state MDSC and Treg levels are estimated based on literature data. The baseline parameter values are estimated using TNBC data, and the parameter ranges are estimated using both TNBC and estrogen-positive/HER2-negative breast cancer data to describe the heterogeneity of HER2-negative patients. Both baseline parameter values and their ranges are listed with references in Supplementary Tables S1–S6.

For each individual as a potential patient, a simulation is performed starting from a single cancer cell with a plausible characteristic parameter set of the patient drawn from our assumed distributions. Due to the lack of patients’ information of their initial tumor diameters at the beginning of the therapy from the clinical trial, an initial tumor diameter is randomly selected for the virtual patient based on our assumed distribution. These preselected initial tumor diameters are then used to calculate the initial tumor volume assuming a spherical tumor as the pre-treatment tumor volume (i.e., preselected initial tumor volume) for the virtual patient. Once the tumor reaches the preselected initial tumor volume, the values of all the species are saved and substituted into the model for further simulations of the therapy of interest. If the tumor has not been able to reach the preselected initial tumor volume, the corresponding individual is considered to not develop a tumor, possibly due to a strong immune response given by the plausible parameter set. These individuals are not included in the post-simulation analysis. The initial conditions and dynamic solutions are calculated using the ode15s solver in MATLAB, and the tumor growth is simulated for 400 days after therapy begins. The absolute tolerance and relative tolerance are set to be 10^–12^ (day) and 10^–6^, respectively. In SimBiology, absolute tolerance controls the largest absolute error allowed for the ODE solver at any step in the simulation, while relative tolerance controls the tolerable error relative to the state vector at each step.

### *In silico* Virtual Clinical Trial and Sensitivity Analysis

For virtual clinical trials, a plausible characteristic parameter set is selected for each potential patient to represent the inter-individual variabilities, such as the cancer killing rate by effector T cells, steady-state Treg and MDSC density in the tumor, antigen binding affinity, cytokine expressions, and tumor mutational burden (TMB), which is measured as the total number of mutations per tumor genomic region and is defined in our model as the number of tumor-specific T cell clones in TDLNs (Li et al., 2016; Yarchoan et al., 2017). The values of selected parameters are assigned using Latin Hypercube Sampling (LHS) based on our estimated distribution and plugged in as input. Among all the simulations, virtual patients who reach the preselected initial tumor volumes are used to calculate the overall response rate and the Partial Rank Correlation Coefficient (PRCC) between post-treatment observations (e.g., end tumor volume, tumor-infiltrating Treg and effector T cell density) and parameters of interest for sensitivity analysis (e.g., cancer cell growth rate, tumor antigen affinity, TMB) (Marino et al., 2008).

### Statistical Analysis

The overall response rate is predicted as the proportion of patients with complete response (CR) or partial response (PR) based on RECIST v1.1, and the 95% Agresti-Coull confidence interval (CI) is estimated based on normal approximation for the binomial distribution. For comparison of model observations between responders and non-responders and that among virtual patients in various therapeutic regimens, Wilcoxon test is performed using ggpubr package in RStudio v1.2 (Kassambara, 2019).

## Results

### Prediction of Entinostat Concentration in Tumor

Figure 2B, demonstrates the simulated plasma concentration of entinostat together with the clinical measurements at dose levels of 2, 4, and 6 mg/m^2^ assuming a body surface area of 1.7 m^2^ (Gore et al., 2008). The simulated peak concentrations are 15.4, 30.8, and 46.3 ng/mL, and the areas under the curve are calculated to be 105.5, 211.0, and 316.8 ng h/mL for doses of 2, 4, and 6 mg/m^2^, respectively. The time t_max_ at the peak concentrations is estimated to be 0.5 h for all doses. To further test the interindividual variability of entinostat concentration, we varied the values of absorption rates and clearance rates of entinostat in the sensitivity analysis. The results are represented by a heatmap below with other parameters of interest, and show that non-linear clearance rate of entinostat has a significant inverse correlation with its anti-proliferative effect on cancer cells.

### Efficacy of Anti-PD-1 Monotherapy and Its Combination With Entinostat

**FIGURE 3:**
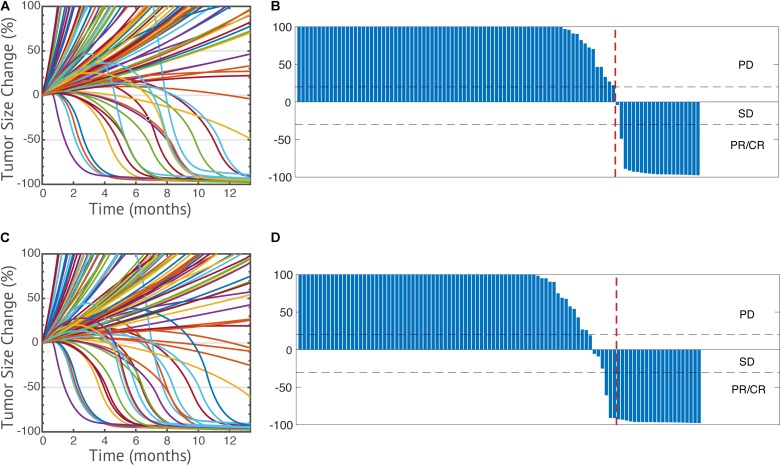
Spider plots of 100 randomly selected virtual patients **(A,C)** and change from baseline in model-predicted tumor diameter assessed by RECIST v1.1 **(B,D)** in anti-PD-1 monotherapy **(A,B)** and its combination with entinostat **(C,D)**. PD, progressive disease; SD, stable disease; PR, partial response; CR, complete response.

The model is first used to simulate the overall anti-tumor response to anti-PD-1 monotherapy in breast cancer. Among the 1500 simulations, 1196 virtual patients reach the preselected initial tumor volume. It should be noted that the ratio 1196/1500 reflects our method of generating the initial conditions and does not reflect the actual fraction of individuals who develop tumors. Thus, it should be considered a methodological detail rather than a reflection of a biological process. The parameter sets of the 1196 virtual patients are saved to simulate anti-tumor response to all the following therapeutic regimens and statistical analysis. Once the tumor diameter has reached its preselected value, 3 mg/kg nivolumab is administered every 2 weeks. The time-dependent percentage change of the tumor size (spider plot) is plotted in Figure 3A, based on RECIST criteria (Eisenhauer et al., 2009). Overall, 265 virtual patients have a partial or complete response (22.2%), and 37 virtual patients have stable disease (3.1%); the remaining 894 patients had progressive disease (74.8%). A waterfall plot of changes from baseline in model-predicted tumor diameter is shown in Figure 3B.

To further investigate the effect of entinostat on the overall response rate and tumor microenvironment, we simulate the overall anti-tumor response to a double combination therapy using 3 mg/kg nivolumab every 2 weeks and weekly doses of 5 mg entinostat. The parameter sets and initial conditions of the same 1196 virtual patients who reached preselected initial tumor volume are saved and used to perform a virtual trial with combination of entinostat and nivolumab. Of the 1196 virtual patients, 320 have a partial or complete response (26.8%), and 52 have stable disease (4.4%), the remaining 824 patients had progressive disease (68.9%). Thus, the predicted increase of the response rate from 22.2% for nivolumab alone to 26.8% for the combination of nivolumab and entinostat. The time-dependent percentage change of the tumor size (spider plot) and the waterfall plot are shown in Figures 3C,D. We can now apply these simulation results to the actual clinical trial in which each dose regimen involves less than 15 patients. By randomly sampling 15 virtual patients 100,000 times, we obtain a 95% percentile bootstrap confidence interval of (6.67%, 46.7%) for our estimate of the overall response rate in the double combination therapy. Thus, even though these results are dependent of the space of parameters for the virtual patients, we note that the predicted confidence interval is very wide.

### Model-Predicted Anti-tumoral Effect in Triple Combination Therapy

**FIGURE 4:**
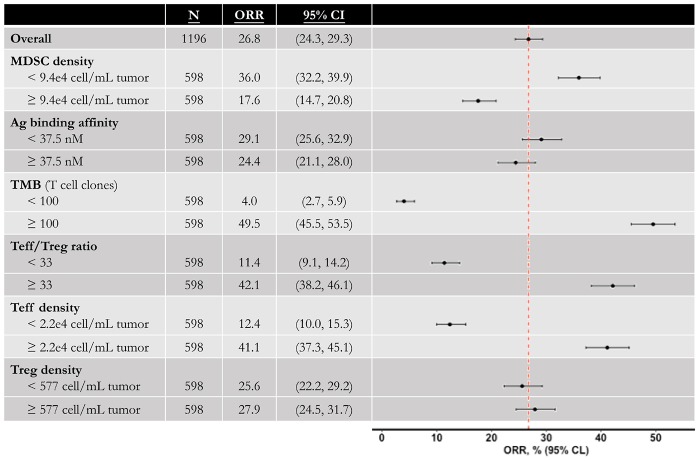
Anti-tumor activity of triple combination therapy in virtual patient cohort. Total 1196 virtual patients in triple combination of entinostat, nivolumab, and ipilimumab are divided into subgroups based on the population medians, and the objective response rates in each subgroup are calculated with 95% Agresti-Coull confidence intervals. MDSC, myeloid-derived suppressor cell; Ag, tumor antigen; TMB, tumor mutational burden (tumor-specific T cell clones in lymph nodes); Teff, effector T cell; Treg, regulatory T cell.

**FIGURE 5:**
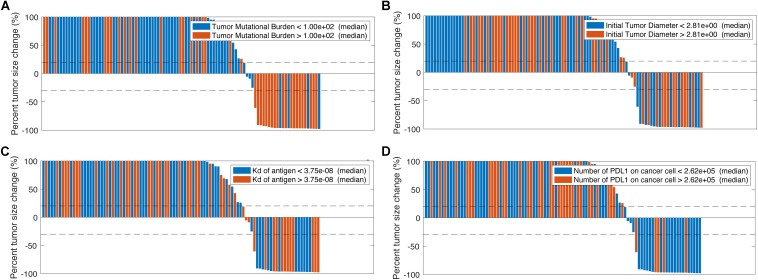
Change from baseline in model-predicted tumor diameter assessed by RECIST v1.1 based on tumor mutational burden **(A)**, initial tumor diameter **(B)**, tumor antigen binding affinity **(C)**, and number of PD-L1 molecules on cancer cells **(D)**.

Now that the efficacy of entinostat on improving anti-tumoral effect of anti-PD-1 monotherapy has been simulated, the model is used to investigate the effect of the addition of anti-CTLA-4 antibody. Four doses of 1 mg/kg ipilimumab are administered every 6 weeks with weekly 5 mg entinostat and 3 mg/kg nivolumab every 2 weeks. While the number of responders remains the same, the mean post-treatment tumor volume is lower than that in the double combination therapy. This slight increase of anti-tumor response is due to the ADCC-mediated Treg depletion by ipilimumab, which significantly increase Teff to Treg ratio in the tumor. The total virtual population is then divided into six subgroups based on their pre-treatment tumor-infiltrating Teff, Treg, and MDSC density, Teff to Treg ratio, TMB, and tumor-specific antigen binding affinity by their medians. The response rates of all subgroups with 95% confidence intervals are shown in Figure 4. The confidence intervals for subgroups MDSC density, TMB, tumor-infiltrating Teff density, and Teff to Treg ratio show significantly different response rates in these subgroups, while those for other subgroups overlap. The 95% percentile bootstrap confidence interval for our estimate of the overall response rate in the triple combination therapy is calculated to be (6.67%, 53.3%) for a sample size of 15, using the same methods in the previous section. 100 out of the 1196 virtual patients are randomly selected to illustrate their changes from baseline in model-predicted tumor diameter with parameters of interest, as shown in Figures 5A–D. While a large portion of responders correspond to patients with high TMB and low PD-L1 expression on cancer cells, antigen binding affinity and initial tumor diameter are evenly distributed between responders and non-responders.

**FIGURE 6:**
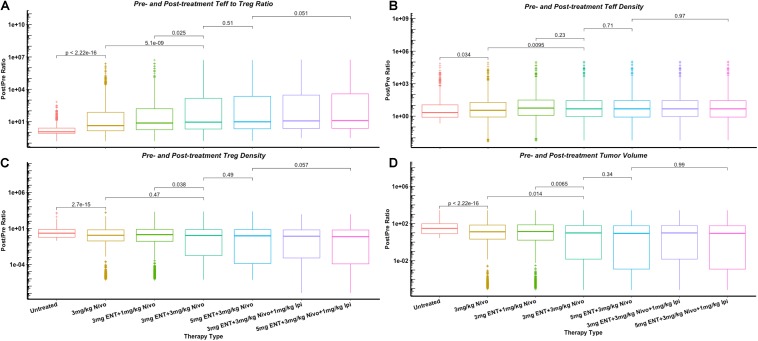
Changes of cell density and tumor volume in dose escalation as post- to pre-treatment ratios, including Teff to Treg ratio **(A)** and their densities in tumor **(B,C)**, and tumor volume **(D)**. *p*-values are calculated using Wilcoxon test.

To further investigate the effect of combination therapy using immune checkpoint inhibitors and the epigenetic modulator, we plot the changes of Teff/Treg density and ratio, and tumor volume as post- to pre-treatment ratio using the five therapeutic regimens from the clinical trial as shown in Figure 6. The significant increase of Teff to Treg ratio when higher doses of nivolumab are administered in the double combination therapy corresponds to the significant increase of tumor-infiltrating Teffs, as nivolumab blocks inhibitory signals on cancer cells and restores Teff functionality. On the other hand, the increases of Teff to Treg ratio by addition of entinostat and ipilimumab correspond more to the decrease of Tregs than to the increase of tumor-infiltrating Teffs. This phenomenon is due to the immune modulation by entinostat that inhibits Treg expansion, as well as the Treg depletion effect by anti-CTLA-4 antibody.

### Anti-tumor Response as Affected by Parameters of Interest

**FIGURE 7:**
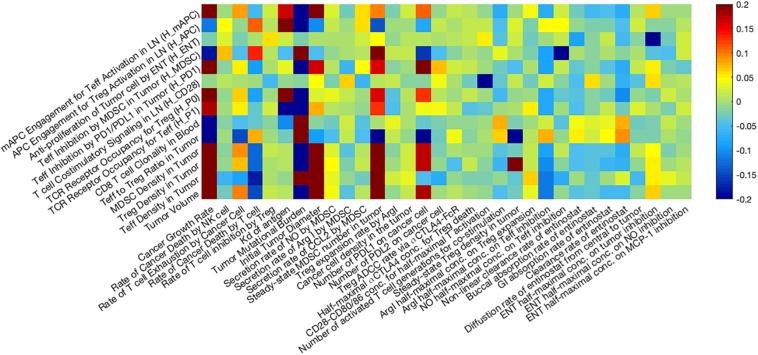
Global uncertainty and sensitivity analysis. Thirty two parameters are assigned using Latin Hypercube Sampling (LHS) based on our estimated distribution, and the Partial Rank Correlation Coefficient (PRCC) between selected post-treatment observations and input parameters are presented as a heatmap. Among 32 parameters, tumor growth rate, T cell exhaustion, cancer killing rate by T cells, TMB, initial tumor diameter, steady-state MDSC density, PD-L1 expression on cancer cells, and inhibitory effect of Arg-I on T cells are significantly correlated with post-treatment tumor volume.

**FIGURE 8:**
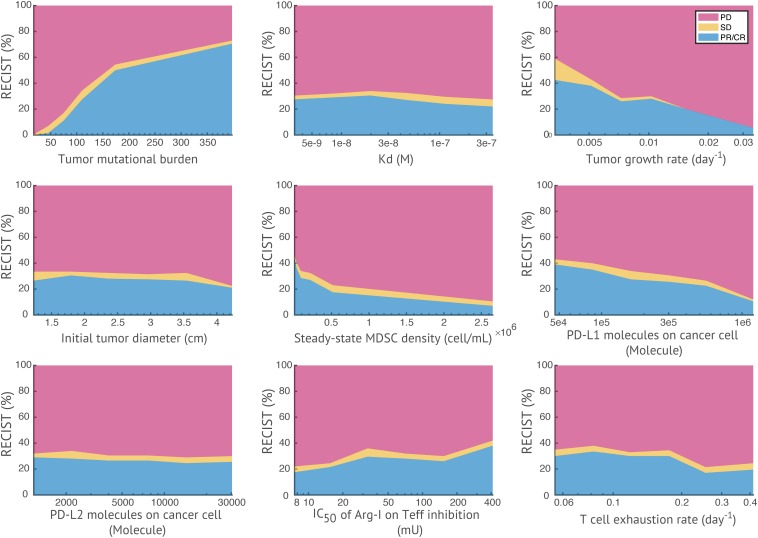
Effects of parameters on patients’ response. For each parameter of interest, virtual patients are sorted by their corresponding parameter values in ascending order, and evenly divided into six subgroups. The response status of each subgroup is plotted against the median parameter values in each subgroup. TMB and effective concentration of Arg-I on Teff inhibition show positive correlations with response rate, while tumor growth rate, steady-state MDSC density, and the number of PD-L1 molecules on cancer cell show negative correlations (i.e., with a >15% increase/decrease of response rates in subgroups).

In Figure 7, a heatmap of global uncertainty and sensitivity analysis shows that among 32 parameters, tumor growth rate, T cell exhaustion, cancer killing rate by T cells, TMB, initial tumor diameter, steady-state MDSC density, PD-L1 expression on cancer cells, and inhibitory effect of Arg-I on T cells are significantly correlated with end tumor volume. The sensitivity of these responses to parameters is further illustrated in Figure 8. In the above simulations based on the reference values of model parameters, we predicted certain response rate at 400 days, e.g., in a combination of nivolumab and entinostat 26.8% have a partial or complete response, 4.4% have stable disease, and 68.9% have progressive disease. However, these percentages are affected by the parameters of the patient cohort, and results of a trial may be different depending on the parameters of the patients within the cohort. Figure 8, illustrates the effects of variation of parameters on the patients’ response according to RECIST criteria for 9 parameters selected from the global sensitivity results. TMB, tumor growth rate, steady-state MDSC density, the number of PD-L1 molecules on cancer cell, and effective concentration of Arg-I on Teff inhibition show strong impacts on tumor size change, which corroborates their statistical significance suggested by PRCC analysis and emphasizes a need for accurate estimation of these parameters for personalized simulations.

### Identification and Performance of Potential Predictive Biomarkers

**FIGURE 9:**
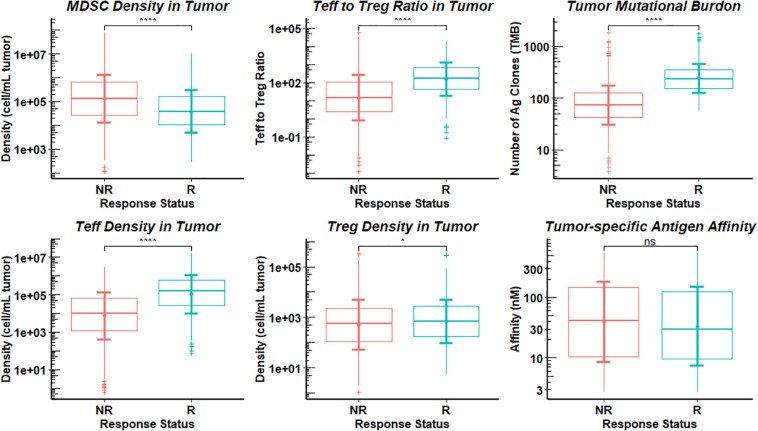
Distributions of Potential Biomarkers in Responders and Non-responders. The response in the 1196 simulations was divided into responders (R) and non-responders (NR), and statistical comparisons are presented between the two groups for pre-treatment observations. Statistical significance is calculated by Wilcoxon test. ^∧^*p*-values ≤ 0.05; ^∧^^∧^^∧^^∧^*p*-values ≤ 0.0001; non-significance (ns), *p*-values > 0.05.

**FIGURE 10:**
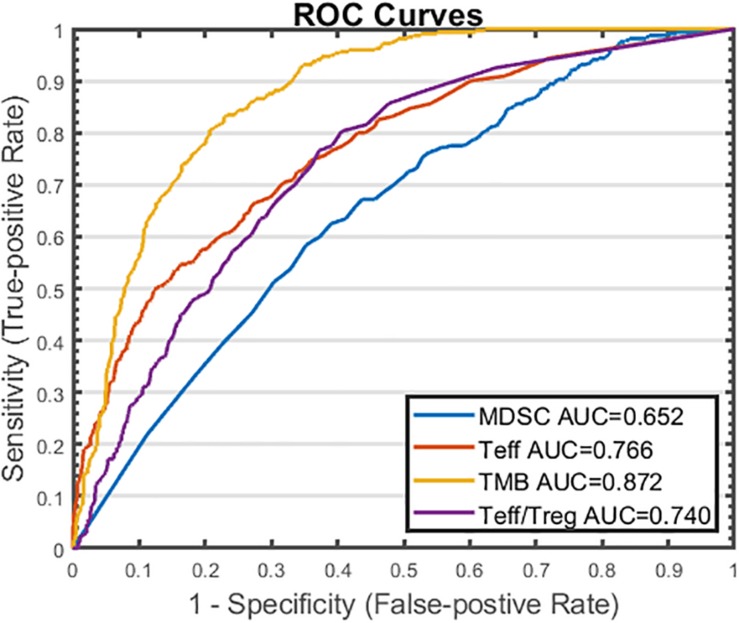
ROC Analysis of Potential Predictive Biomarkers in Triple Combination Therapy. Cutoff values are selected based on the range of pre-treatment amounts of myeloid-derived suppressor cell (MDSC) density, effector T cell (Teff) density, tumor mutational burden (TMB), and Teff to regulatory T cell (Treg) ratio. For each cutoff value, response status (R vs. NR) is predicted for each virtual patient by comparing the pre-treatment amount of the potential biomarker to the cutoff value. Sensitivity (true positive rate) is plotted against 1 – specificity (true negative rate) for each biomarker. R, responders; NR, non-responders.

From sensitivity analysis and overall response table presented above, we identify several potential predictive biomarkers for the triple combination therapy in this virtual clinical trial. As shown in Figure 9, the distributions of pre-treatment tumor-infiltrating Teff and Treg density, Teff to Treg ratio, and TMB are significantly higher in responders when compared with those in non-responders, while MDSC density is significantly higher in non-responders, possibly due to its strong immune-suppressive activity in the tumor microenvironment. We further investigate the performance of these potential biomarkers on prediction of anti-tumor response to the triple combination therapy through binary classification. As shown in Figure 10, the Sensitivity and 1-Specificity values from each cutoff were plotted as ROC curves. TMB, tumor-infiltrating Teff and Teff to Treg ratio have higher AUCs (0.872, 0.766, and 0.740, respectively) than intra-tumoral MDSC density (0.652), further implicating their potential to be predictive biomarkers for this triple combination regimen.

## Discussion

Based on our previously published QSP model and our first attempt to make personalized predictions of anti-tumor response to immunotherapy using immune checkpoint inhibitors (Milberg et al., 2019; Wang et al., 2019), a more recent model with reduced number of ODEs has been published by Jafarnejad et al. (2019) through simplification of certain processes, such as T cell trafficking and the kinetics of T cell priming in the lymph nodes. In this study, we present a further developed model to: modularize each species of interest and individually calibrate the modules based on literature data; add a MDSC module; investigate the mechanisms of action for the epigenetic modulator and checkpoint inhibitors with limited and/or controversial preclinical results; and conduct a virtual clinical trial of a combination therapy using anti-PD-1, anti-CTLA-4 antibodies and an epigenetic modulator, entinostat. The modularized model can be readily reproduced, and additional modules can be added in future studies if the dynamics of other molecular and cellular species are of interest.

While T cell trafficking equations and the PK/PD module of checkpoint inhibitors remained unchanged compared to our previous models (Jafarnejad et al., 2019; Wang et al., 2019), we adopted a new mechanism of T cell activation by mature APCs (mAPCs) (Lever et al., 2014). Tumor antigens are released from dying cancer cells and transported to TDLNs. Virtual patients with higher TMB would generate higher levels of tumor antigens, which can be recognized by higher levels of tumor-specific naïve T cells in TDLNs. Activation of tumor-specific naïve T cells in TDLNs is modeled by a kinetic proofreading module with limited signaling based on TMB, binding affinity of tumor antigens and concentration of mAPCs (Lever et al., 2014). The activated T cells proliferate through a calculated number of generations based on T cell receptor signaling, co-stimulatory signaling, and cytokine signaling before returning to quiescence, while they simultaneously differentiate into effector T cells (Marchingo et al., 2014). In addition, simulations also start from a single cancer cell to capture the initial conditions at the preselected initial tumor volumes. This way, we take into account the virtual patients whose adaptive immune response is strong enough to eliminate the cancer cells before developing into a tumor. Using this method, we no longer observe the strong correlation between tumor volume and initial tumor diameter (Figure 8) as we did in previous study (Wang et al., 2019). By starting model simulation from a single cancer cell, larger initial tumor size is more likely to acquire higher number of pre-treatment tumor-infiltrating T cells, which results in a similar T cell density to smaller sizes. Thus, anti-tumor response is less dependent on its initial size.

According to our model analysis, a positive correlation between PD-L1 expression on cancer cell and end tumor volume is observed. That is, patients with small number of PD-L1 molecule on cancer cell are likely to have strong anti-tumor response. This is due to our assumption that Teff function is not inhibited by cancer cells without PD-L1 expression, opposite to our previous model, where we assume that Teffs are inhibited by other inhibitory pathways if not by PD-L1 on cancer cells (Wang et al., 2019). Both of the assumptions aim to explain the correlation between PD-L1 status and anti-tumor response observed in clinical trials (Vikas et al., 2018). To further investigate the implication of PD-L1 positivity in patients with breast cancer, its expression on both cancer cells and immune cells should be implemented in future studies with appropriate assumptions of their functionality based on clinical evidence (Marra et al., 2019; Matikas et al., 2019). For example, PD-L1 expression on macrophages plays an important role in macrophage polarization and antitumor cytokine secretion (Hartley et al., 2018). In addition, PD-L1 expression on cancer cells shows correlations with tumor metastasis and suppression of effector T cells, which is regulated by epithelial-to-mesenchymal transition of cancer cells (Chen et al., 2014; Terry et al., 2017). Therefore, a macrophage module can be added to investigate the interactions between macrophages and cancer cells and the resulting effects on the tumor microenvironment (Mahlbacher et al., 2018; Li et al., 2019; Zhao et al., 2019).

In our previous model (Wang et al., 2019), we suggested that the MDSC level in the tumor was significantly related to anti-tumor response to combination checkpoint blockade therapy, assuming that the inhibition of effector T cells by MDSCs was mainly dependent on their checkpoint expression and that intratumoral Treg level remained a constant fraction of MDSCs. In the present model, we further expand the mechanisms of MDSCs to include both the secretion of Arg-I and NO by MDSCs, which inhibit Teff cytotoxicity and induce Treg expansion, and CCL2 secretion by breast cancer cells, which facilitates MDSC recruitment into the tumor. As shown in the sensitivity analysis (Figure 7), the addition of detailed MDSC mechanisms does not lead to an overestimated inhibition of the immune response, as the efficacy of the combination therapy is significantly correlated to not only MDSC-related parameters but also to other immune-suppressive factors.

To study the efficacy of entinostat, we proposed a pharmacokinetic model to estimate the transport parameters based on the plasma concentration measured by Gore et al. (2008). The simulated peak concentration and area under the plasma concentration curve for different doses are compared with other published PK analysis data of entinostat (Ryan et al., 2005; Gojo et al., 2007; Kummar et al., 2007). Although most of our simulated concentrations fall within the range of their clinically measured values, the small sample sizes and the large differences in means and ranges reported in all the four studies suggest that additional clinical measurements are needed to improve our prediction of entinostat concentration in patients with breast cancer. For pharmacodynamics of entinostat, it is assumed to inhibit proliferation of breast cancer cells and the cytokine secretion by MDSCs, reversing their inhibitory effects on T cell subsets. Interestingly, the effective concentrations of entinostat on its anti-proliferative activity are dependent on the subtypes (i.e., HS-578t, MCF-7, ZR-75, and SKBR3), and it also reduces cell viability in some subtypes of breast cancer, including MCF-7, ZR-75, and SKBR3 cells (Lee et al., 2001). The discrepancy of the efficacy of combination therapy using immune checkpoint inhibitors and epigenetic modulator among different cancer types might result from this difference in effective concentrations (Gallagher et al., 2017).

For mechanism of action of anti-CTLA-4 antibody, it was assumed in our previous model that the efficacy of anti-CTLA-4 therapy observed in clinical trials is mainly mediated by blocking CTLA-4 and CD80/86 interactions and thus restoring co-stimulatory signaling in T cell activation in TDLNs (Wei et al., 2018). In addition, recent studies suggest that Fc domain of the anti-CLTA-4 antibody is required for efficacy in mouse tumor models, which is critical to induce Fc-mediated depletion of regulatory T-cells (Arce Vargas et al., 2018; Ingram et al., 2018; Tang et al., 2018). However, this newly proposed mechanism of anti-CTLA-4 antibody has shown controversial results from clinical studies (Romano et al., 2015; Sharma et al., 2019). While both mechanisms are implemented in the present model to investigate their roles in anti-tumoral activity in breast cancer, the major mechanism of action of anti-CTLA-4 antibody that contributes to its efficacy has yet to be determined by future studies, which might also be cancer type-dependent.

By conducting a prospective virtual clinical trial, we aim to make predictions of the anti-tumor activities and biomarkers for an ongoing trial that has not yet been completed. Starting from the nivolumab monotherapy, we predict the response rate of 1196 virtual patients with breast cancer. A 22.2% response rate is predicted given our set of parameters of interest with assumed distributions. Based on our assumptions on the mechanisms of action, our predicted response rate falls within the reported range of response rate of anti-PD-1 monotherapy using pembrolizumab in patients with TNBC or estrogen-positive/HER2-negative metastatic breast cancer (Emens, 2018; Planes-Laine et al., 2019), which demonstrate the ability of the present model to perform virtual clinical trials and make reasonable qualitative predictions on anti-tumor response. When combined with entinostat, the response rate of checkpoint therapy increases to 26.8%. However, it is challenging to quantify the improvement of anti-tumor response to checkpoint blockade therapy by the addition of entinostat, since the PK parameters and the effective concentrations are only roughly estimated for entinostat. Although combination therapy of entinostat and anti-PD-1 antibody has shown promising results in patients with anti-PD-1-resistant melanoma and non-small cell lung cancer, the effect of cancer type and patients’ characteristics on the improved efficacy has yet to be determined (Agarwala et al., 2018; Hellmann et al., 2018). Furthermore, the simulations show that the addition of four doses of anti-CTLA-4 antibody ipilimumab does not significantly improve the performance of the combination therapy, even though Teff to Treg ratio is significantly increased due to ADCC. This result is also suggested by our previous model (Wang et al., 2019), and higher doses of the ipilimumab might be required to improve the T cell activation and thus anti-tumor response; however, in the clinic, higher doses of ipilimumab are limited by toxicity. Overall, the model suggests that TMB, tumor-infiltrating Teff density, and Teff to Treg ratio can be predictive biomarkers in this triple combination therapy. The efficacy of all the tested therapies shows strong correlation with these model observations, which is also supported by their clinical significance in anti-tumor response and overall survival in breast cancer (Adams et al., 2014; Asano et al., 2016; Takada et al., 2018; Thomas et al., 2018).

Notably, the predictions of anti-tumor response and predictive biomarkers are strongly affected by our assumptions on mechanism of action for all therapeutics and distribution of physiological parameters for virtual patient cohort (Cassidy and Craig, 2019). The expected response rate of the ongoing clinical trial simulated in this study, as suggested by the 95% percentile bootstrap confidence intervals, could fall into a wide range. Due to the variations of selection criteria and settings in clinical trials, the distribution of patient parameters can be largely different and in fact only a few of the parameters that are necessary as inputs for the model are clinically measured; most of the parameters remain unknown for each particular patient or a cohort that results in uncertainty of model predictions. For example, the generally lower overall response rate reported in previously treated TNBC patients might result from their changes of physiological parameters in previous therapy when compared with previously untreated patients (Adams et al., 2019a, b). In this case, our model proposes to consider high TMB, tumor-infiltrating Teff density, and Teff to Treg as potential biomarkers, which might improve anti-tumor response in previously treated patients (Alva et al., 2019). Importantly, ongoing clinical trials may provide insights on the effect of entinostat and ipilimumab on the immune system and resistance mechanism in breast cancer development, which would allow us to make step-by-step modification of the model and its parameters and improve its predictive power (Pitt et al., 2016; Darvin et al., 2018; Eladdadi et al., 2018; Mahlbacher et al., 2019). Our goal is to understand the dynamic interactions between drugs and the immune system in cancer as a whole, to update our assumptions on drug/tumor-immune dynamics through comparison between model predictions and clinical observations, and thereby to guide drug development and clinical trial design (Cheng et al., 2017; Nijsen et al., 2018; Bai et al., 2019; Bradshaw et al., 2019).

## Data Availability Statement

The authors confirm that the data supporting the findings of this study are available within the article and the Supplementary Material. MATLAB scripts for model and data generation for this study will be made available by the corresponding author, without undue reservation, to any qualified researcher on request.

## Author Contributions

HW, AP, RC, and ET designed and planned the project. AP supervised the project and critically edited the manuscript. MJ, RS, HW, and SR built and modified the model structure. HW performed all simulations, analyzed the simulation data, and prepared a draft of the manuscript. EJ, VS, ET, and RC critically revised the manuscript. All authors have read and approved the final manuscript.

## Conflict of Interest

RC has received research grants to institution from Novartis, Puma Biotechnology, Merck, Merrimack, Genentech, Macrogenics, and travel expenses from Genentech. The remaining authors declare that the research was conducted in the absence of any commercial or financial relationships that could be construed as a potential conflict of interest.

## Supplementary Material

FIGURE S1 Antiproliferative effect of entinostat (ENT) on Breast Cancer Cell. 1-Hill function of inhibitory effect of ENT vs. its concentration **(A)**, and number of cancer cells vs. time **(B)**. Experimental data is from Lee et al. (2001) (PMID: 11221885). WT, wild type. Exp, experiment. Sim, simulation.

FIGURE S2 Arginase I and nitric oxide expression. The error bars represent the range of arginase I **(A)** and nitric oxide **(B)** in virtual patient cohort, fitted to experimental data from Serafini et al., 2008 (PMID: 18593947).

FIGURE S3 MCP-1/CCL2 Expression **(A)** and Recruitment of MDSC **(B)**. Range of CCL2 expression is fitted to data from Dutta et al. (2018) (PMID: 29594759), and effective concentration of CCL2 on recruitment of MDSC into the tumor is optimized to match the migration index reported by Huang et al., 2007 (PMID: 17257744).

TABLE S1 Model Compartment Name, Capacity, Unit, and Description.

TABLE S2 Model Parameter Name, Value, Unit, and Description.

TABLE S3 Model Reaction, Reaction Rate, and Description.

TABLE S4 Model Algebraic Equations (Model Rules).

TABLE S5 Model Species Name, Initial Amount, Unit, Location, and Description.

TABLE S6 Model Events.

TABLE S7 Model Parameters used in MDSC Module.
