# Supplementary material for: Conducting a Virtual Clinical Trial in HER2-Negative Breast Cancer Using a Quantitative Systems Pharmacology Model With an Epigenetic Modulator and Immune Checkpoint Inhibitors
Source: Front Bioeng Biotechnol. 2020 Feb 25;8:141. doi: 10.3389/fbioe.2020.00141 (PMC7051945; doi:10.3389/fbioe.2020.00141)
Supplement: FIGURE S1 — Antiproliferative effect of entinostat (ENT) on Breast Cancer Cell. 1-Hill function of inhibitory effect of ENT vs. its concentration (A), and number of cancer cells vs. time (B). Experimental data is from Lee et al. (2001) (PMID: 11221885). WT, wild type. Exp, experiment. Sim, simulation. [file Data_Sheet_1.zip › Wang et al - Supplementary.docx]

Supplementary Material

A

B

**Figure S1.** **Antiproliferative Effect of Entinostat (ENT) on Breast Cancer Cell.** 1-Hill function of inhibitory effect of ENT vs. its concentration (A), and number of cancer cells vs. time (B). Experimental data is from Lee et al. 2001 (PMID: 11221885). WT, wild type. Exp, experiment. Sim, simulation.

**
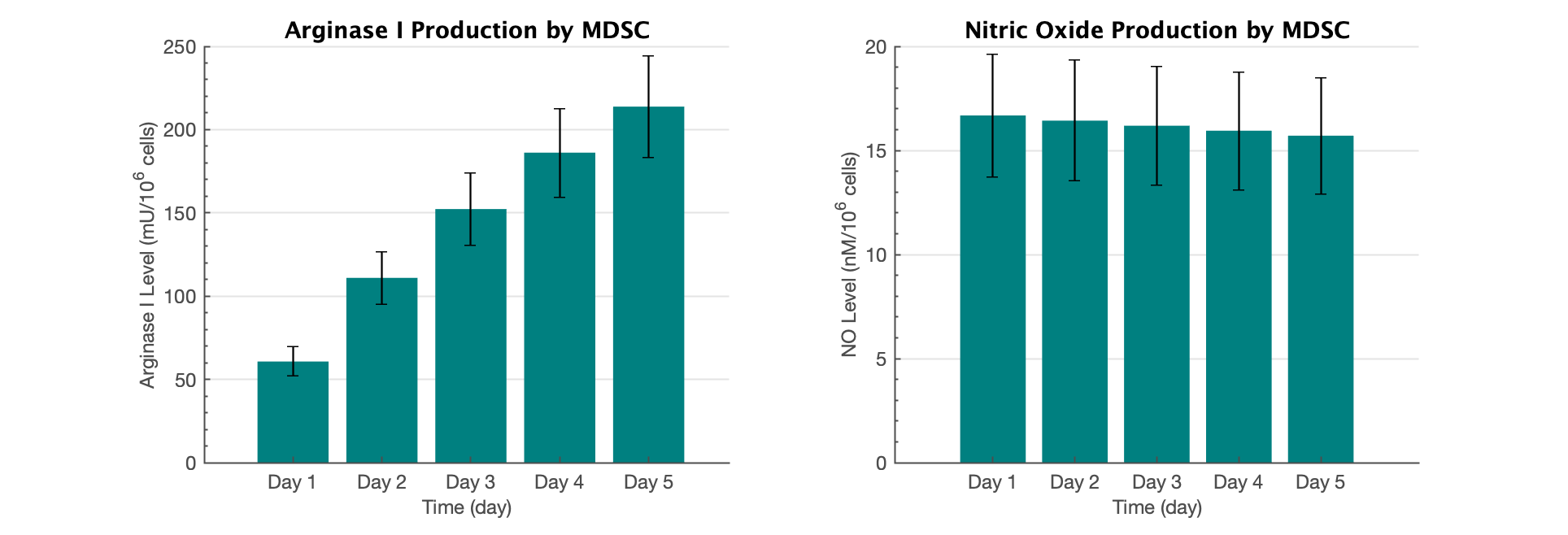
**

B

A

**Figure S2. Arginase I and Nitric Oxide Expression.** The error bars represent the range of arginase I (A) and nitric oxide (B) in virtual patient cohort, fitted to experimental data from Serafini et al. 2008 (PMID: 18593947).

**
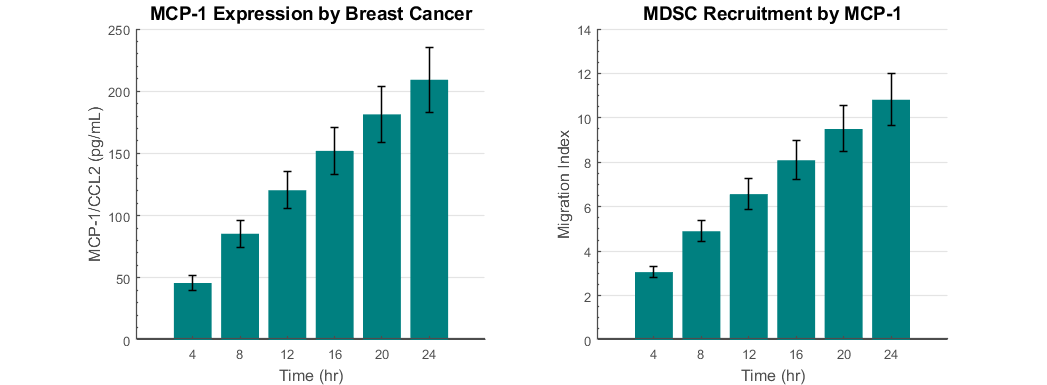
**

A

B

**Figure S3. MCP-1/CCL2 Expression (A) and Recruitment of MDSC (B).** Range of CCL2 expression is fitted to data from Dutta et al. (PMID: 29594759), and effective concentration of CCL2 on recruitment of MDSC into the tumor is optimized to match the migration index reported by Huang et al. 2007 (PMID: 17257744).
